# *PHO15* genes of *Candida albicans* and *Candida parapsilosis* encode HAD-type phosphatases dephosphorylating 2-phosphoglycolate

**DOI:** 10.1093/femsyr/foy112

**Published:** 2018-10-10

**Authors:** Eliška Kročová, Sylva Neradová, Rudolf Kupcik, Sylva Janovská, Zuzana Bílková, Olga Heidingsfeld

**Affiliations:** 1 Department of Biological and Biochemical Sciences, Faculty of Chemical Technology, University of Pardubice, 532 10 Pardubice, Czech Republic; 2 Gymnasium, Pardubice, Mozartova, 530 09 Pardubice, Czech Republic; 3 Institute of Organic Chemistry and Biochemistry, Academy of Sciences of the Czech Republic, 166 10 Prague, Czech Republic

**Keywords:** *Candida albicans*, *Candida parapsilosis*, phosphatase, HAD-family, 2-phosphoglycolate, *PHO15*

## Abstract

Most of the phosphatases of human fungal pathogens *Candida albicans* and *C. parapsilosis* have never been experimentally characterised, although dephosphorylation reactions are central to many biological processes. *PHO15* genes of these yeasts have been annotated as the sequences encoding 4-nitrophenyl phosphatase, on the basis of homology to *PHO13* gene from the bakers’ yeast *Saccharomyces cerevisiae.* To examine the real function of these potential phosphatases from *Candida* spp., CaPho15p and CpPho15p were prepared using expression in *Escherichia coli* and characterised. They share the hallmark motifs of the haloacid dehalogenase superfamily, readily hydrolyse 4-nitrophenyl phosphate at pH 8–8.3 and require divalent cations (Mg^2+^, Mn^2+^ or Co^2+^) as cofactors. CaPho15p and CpPho15p did not dephosphorylate phosphopeptides, but rather hydrolysed molecules related to carbohydrate metabolism. The preferred substrate for the both phosphatases was 2-phosphoglycolate. Among the other molecules tested, CaPho15 showed preference for glyceraldehyde phosphate and ß-glycerol phosphate, while CpPho15 dephosphorylated mainly 1,3-dihydroxyacetone phosphate. This type of substrate specificity indicates that CaPho15 and CpPho15 may be a part of metabolic repair system of *C. albicans* and *C. parapsilosis*.

## Teaser

PHO15 genes of *Candida albicans* and *C. parapsilosis* encode HAD-type phosphatases, which have not been characterised as yet and dephosphorylate 2-phosphoglycolate.

## INTRODUCTION

The yeasts *Candida albicans* and *C. parapsilosis* are among the most clinically relevant *Candida* species that represent a serious threat to individuals, whose immune system has been weakened, mucosal barriers damaged or whose natural microflora has been disturbed. Until the 1950s, *Candida* had only rarely been associated with diseases (Casadevall and Pirofski 2015). However, advanced medical treatment such as chemotherapy, organ transplantation or wide use of antibiotics significantly improved survival rates for critically ill patients. As a consequence, the number of patients at risk has increased and infections caused by pathogenic *Candida* species have become a serious public health problem (Pfaller and Castanheira 2016). Superficial infections, e.g. oral or vaginal thrush, can be usually cured by antimycotics available to date (Bondaryk, Kurzątkowski and Staniszewska 2013). However, disseminated invasive candidiasis, which affects normally sterile body fluids and tissues, is often refractory to treatment and is associated with high mortality rates (Berman 2012; Bilir *et al.*^2015^). *Candida albicans* and *C. parapsilosis* are among causative agents of nosocomial bloodstream infections that spread through indwelling medical devices or through the hands of health care personnel (Guinea 2014; Pfaller and Castanheira 2016).

The genomes of several *Candida* species including *C. albicans* and *C. parapsilosis* have been sequenced (Butler *et al.*^2009^; Jones *et al.*^2004^) and *C. albicans*, which is the most frequent human fungal pathogen, has been extensively studied. The number of studies focusing on *C. parapsilosis* has recently increased as well. Availability of *Candida* genome sequences accelerated molecular analysis of virulence traits and overall lifestyle of these fungi (Polke, Hube and Jacobsen 2015). Despite the rapid advancement, a large proportion of open reading frames in pathogenic *Candida* species have not been experimentally characterised and their function is being inferred from electronic annotation, relying mostly on similarities with orthologous genes in baker´s yeast *Saccharomyces cerevisiae*. This is also the case of *PHO15* genes of *C. albicans* and *C. parapsilosis* (C1_07230W_A and CPAR2_206650, respectively). Proteins encoded by these genes, CaPho15p and CpPho15p, have been annotated as alkaline phosphatases, more specifically as para-nitrophenyl phosphatases, according to a common compound used for colorimetric assay of phosphatase activity. However, they have never been experimentally characterised. The annotation is based on homology to alkaline phosphatase Pho13p of *S. cerevisiae* (ScPho13p), for which dephosphorylation of para-nitrophenylphosphate (pNPP) has been experimentally confirmed (Tuleva, Vasileva-Tonkova and Galabova 1998).

Catalytic mechanism of alkaline phosphatases usually involves conserved serine or threonine residue acting as a nucleophile that is transiently phosphorylated in the course of catalysis (O’Brien and Herschlag 2002; Pabis and Kamerlin 2016). However, protein sequence analysis of ScPho13p and the both *Candida* Pho15p suggested that they differ from majority of well-known alkaline phophatases and categorise into haloacid dehalogenase (HAD) superfamily. Despite the name that has been derived from archetypal enzyme haloacid dehalogenase of *Pseudomonas* species, this superfamily includes enzymes catalysing phosphoryl or carbon transfer reactions on a diverse range of substrates, and also a large number of enzymes of unknown function (Burroughs *et al.*^2006^; Motosugi, Esaki and Soda 1982).

The hallmark of HAD enzymes is the DxD signature, two aspartic residues separated by one amino acid and coordinating Mg^2+^ ion. The first of the aspartates acts as a nucleophile and forms aspartylphosphate intermediate during the catalysis (Burroughs *et al.*^2006^). Free phosphate is released and catalytic aspartate regenerated upon subsequent nucleophilic attack by a water molecule that has been deprotonated by the second aspartate (Asp + 2) of the DxD motif. Mg^2+^ facilitates correct positioning of substrates in the active site and stabilises the transition state (Seifried, Schultz and Gohla 2013). The catalytic aspartates are a part of a motif I, which is one of four conserved motifs constituting the active sites of HAD family phosphatases. Motif II contains Ser/Thr after a stretch of hydrophobic amino acids. The role of Ser/Thr residue is formation of hydrogen bond with the phosphoryl group of a substrate. Motif III is poorly conserved, but the key residue is Lys that stabilises the reaction intermediate. Motif IV, (G/S)(D/S)x3–4(D/E), provides two additional Asp or Asp and Glu residues that are involved in coordination of Mg^2+^, together with aspartates of motif I (Seifried, Schultz and Gohla 2013).

Biochemical and structural characterisation of 13 recombinant HAD-like phosphatases of *S. cerevisiae* has been recently published, and ScPho13p has been included to this panel (Kuznetsova *et al.*^2015^). All of these enzymes required divalent cations (Mg^2+^, Mn^2+^, Co^2+^) and pH 6.5–7.5 for their activity. ScPho13p hydrolysed pNPP, but did not dephosphorylate phosphopeptides. This is in contrast to results obtained with authentic ScPho13p isolated from yeast lysates, which was reported to dephosphorylate phosphoserines in casein and histone II-A and displayed highest activity at pH 8.2 (Tuleva, Vasileva-Tonkova and Galabova 1998). Recombinant ScPho13p instead dephosphorylated 2-phosphoglycolate, glycerol-2-phosphate or phosphoenolpyruvate (Kuznetsova *et al.*^2015^). Since ScPho13p was found to be a part of yeast metabolite repair system acting on potentially toxic substrates that arise as side products of glycolytic metabolism, preferential dephosphorylation of small molecules by this enzyme seems more likely (Collard *et al.*^2016^). The discordant data nonetheless called for further investigation.

Current knowledge of the HAD superfamily phosphatases of *C. albicans* and *C. parapsilosis* is restricted to transcription regulation of *PHO15* genes. Global transcription profiling studies of *C. albicans* placed *CaPHO15* in a subset of 24 genes that were transcriptionally induced by multiple types of stresses, constituting so-called core stress response (Enjalbert *et al.*^2006^), and in a group of genes involved in regulation of switch from default, white cells to opaque, mating competent cells (Hnisz, Schwarzmüller and Kuchler 2009). The data on *CpPHO15* are sparse. This gene was slightly upregulated in a *C. parapsilosis* mutant lacking transcription factor Bcr1p responsible for regulation of biofilm formation (Ding *et al.*^2011^).

The present study aims at characterisation of CaPho15p and CpPho15p on protein level. The both phosphatases were expressed in *Escherichia coli*, purified and subjected to substrate specificity testing, which showed clear preference for small molecules related to carbohydrate metabolism over phosphopeptides.

## MATERIALS AND METHODS

### Construction of PHO15 expression vectors

*Candida albicans* HE169 and *C. parapsilosis* P69 that were originally isolated from blood and ear, respectively, were obtained from the mycological collection of the Faculty of Medicine, Palacky University, Olomouc, Czech Republic. The yeasts were cultivated overnight in the YPD medium (yeast extract-peptone-dextrose; Sigma-Aldrich) and harvested by centrifugation at 4000 g for 10 min. Then, the DNA from the yeasts was isolated as described by Hoffman and Winston (1987). *PHO15* sequences were amplified using the primers 5΄-gccgcgcggcagccaGTCAATTAAAATTACTTCTAAAGA-3΄ and 5΄- gtcatgctagccataCTAATTGTTAAGCTCATGGA-3΄ for *C. albicans*; gccgcgcggcagccatATGTCAGTAAAAATAACTG and gtcatgctagccataTCAATGGGTAAATTCGTA for *C. parapsilosis*. The sequences complementary to *CaPHO15* and *CpPHO15* are capitalised. PCR products were inserted into NdeI site of pET28b vector using the InFusion HD Cloning Kit (Clontech). The resulting vectors pET28-albi-Pho15 and pET28-para-Pho15 enabled expression of CaPho15p and CpPho15p with His-tags at the N-termini.

### Bacterial expression of CaPho15p and CpPho15p

*Escherichia coli* BL21(DE3) transformed either with pET28-albi-Pho15 or with pET28-para-Pho15 were cultivated in LB medium supplemented with kanamycin in a rotation shaker at 37°C up to OD_600_ = 0.8. Then, Pho15p expression was induced by addition of isopropyl-β-D-thiogalactopyranoside (IPTG; final concentration 1 mmol/l). The culture was incubated for another 3 h. The cells were harvested by centrifugation at 4000 g for 10 min.

### Isolation and solubilisation of inclusion bodies

Bacterial cells were resuspended in TN buffer (100 mM Tris-HCl, 150 mM NaCl, pH 7.4) containing lysozyme (1 mg/g of wet biomass). The mixture was stirred at room temperature for 30 min and frozen at –20°C. After thawing, the mixture was treated by sonication and the resulting cell lysate was centrifuged at 9000 g for 10 min. The sediment containing inclusion bodies was washed twice with 100 mM Tris-HCl pH = 7.0, 2 M urea, 5 mM EDTA, 2% Triton X-100. The inclusion bodies were solubilised in 50 mM Tris-HCl pH 8.0, 8 M urea, 1 mM glycine, 1 mM EDTA, 100 mM β-mercaptoethanol and dialysed against 20 mM Tris-HCl, 150 mM NaCl, 100 mM MgCl_2_ pH 7.0. Alternatively, the inclusion bodies were solubilised in 60% acetic acid (v/v) and dialysed against distilled water.

### Co-expression of CaPho15 and CpPho15 with chaperones

*Escherichia coli* BL21(DE3) were transformed with pG-KJE8 vector (TaKaRa) conferring chloramphenicol resistance and containing sequences for expression of five different chaperones. Transformants were cultivated in LB broth supplemented with 20 μg/ml chloramphenicol, collected by centrifugation and made competent using the protocol of Sambrook and Russell (2001). These competent cells were then transformed either with pET28-albi-Pho15 or with pET28-para-Pho15. Transformants were selected using LB-agar containing 20 μg/ml chloramphenicol and 40 μg/ml kanamycin.

The transformants were inoculated into LB broth containing 20 μg/ml chloramphenicol and 40 μg/ml kanamycin. 0.5 mg/ml L-arabinose and 5 ng/ml tetracycline were added to induce chaperone expression. The culture was incubated in a rotation shaker at 37°C up to OD_600_ = 0.4–0.6. Then, IPTG was added to the final concentration of 1 mM/l. The culture was incubated for another 3 h and harvested by centrifugation at 4000 g for 10 min.

### Purification of recombinant phosphatases

Five millilitre of Ni-NTA Agarose (Qiagen) was equilibrated with the wash buffer (10 mM imidazole in distilled water). After centrifugation at 3000 g for 5 min, the supernatant was removed. Subsequently, solubilised inclusion bodies after dialysis with the addition of imidazole (final concentration 10 mM/l) were added to the Ni-NTA Agarose and the suspension was incubated for 1 h on a rotator at a room temperature. After the incubation, the mixture was centrifuged again and the supernatant was collected. The Ni-NTA Agarose was washed with the wash buffer five times. The phosphatases were left immobilised on the Ni-NTA Agarose. Suspension of Ni-NTA Agarose with bound Pho15p in the wash buffer was stored at 4°C as a stock solution. The activity of the phosphatases remained stable up to 3 months.

Phosphatases were eluted from Ni-NTA Agarose either with 300 mM imidazole solution or with a 50 mM Bis-Tris Propane, 4 mM MgCl_2_ buffer of pH 9, 9.5 or 10.4. Alternatively, phosphatases were released from the Ni-NTA Agarose by cleavage of the His-Tag: 2 U of thrombin in Tris-HCl pH 9.5, 50 mM NaCl, 10 mM MgCl_2_ were added and the mixture was incubated for 16 h on rotator at room temperature.

### Assays of phosphatase activity

Activity of Pho15p enzymes was measured using the chromogenic substrate pNPP. For free Pho15p, the solubilised Pho15p after dialysis was mixed with 2x concentrated optimal buffer (see below) and with 15.2 mM pNPP in ratio 1:2:1. The sample was incubated for 20 min on an end-over-end rotator. After the incubation, 100 μl of the sample was pipetted to a microtiter plate. After addition of 18 μl of 6 M NaOH, absorbance of the sample was measured at 405 nm.

To perform the assay using immobilised phosphatases, ∼100 μg of Pho15p bound to Ni-NTA resin via the His-tag was mixed with 300 μl of a 50 mM Tris-HCl buffer containing 10 mM MgCl_2_ and 1.77 mM pNPP. The mixture was incubated for 20 min on an end-over-end rotator at a room temperature and centrifuged for 30 s at 9660 g. The supernatant (100 μl) was pipetted to a microtitre plate. After addition of 18 μl of 6 M NaOH, absorbance of the samples was measured at 405 nm.

In order to determine pH optimum of the phosphatases, pH of the buffer was adjusted to 6, 6.5, 7, 7.5, 7.75, 8, 8.3, 8.5, 9 and 9.5. To determine optimum Mg^2+^, Mn^2+^ and Co^2+^ concentration, 50 mM Tris-HCl buffer was adjusted to pH 8.3 for CaPho15p and to pH 8.0 for CpPho15p, and MgCl_2_ was added to final concentration ranging from 0.01 to 100 mM. For the Mn^2+^ and Co^2+^ optima, the NaOH was not included due to the precipitation of the ions. Optimum temperature was determined using 50 mM Tris-HCl buffer.

To determine the relative values of the absorbance, the replicates were arithmetically averaged, the average was reduced by the arithmetic average absorbance of the adequate blank and the highest value was set equal to 1. The other values were calculated accordingly. To compare separate measurements, previous step was repeated, except the subtraction of the blank average as its value was already included, and using the gained averages from the separate measurements. Standard deviation was determined according to the following formula:

\begin{equation*}
s = \sqrt {\frac{{\mathop \sum \nolimits_{i = 1}^N {{\left( {{v_i} - \bar{v}} \right)}^2}}}{{N - 1}} - \frac{{\mathop \sum \nolimits_{i = 1}^N {{\left( {{b_i} - \bar{b}} \right)}^2}}}{{N - 1}}}
\end{equation*}

where s is the standard deviation, N is the overall number of values, i is an index of a given value of absorbance in the sample, v is the given value, $\bar{v}$ is the arithmetic average of the adequate values for sample, b is the value of absorbance in the blank and $\bar{b}$ is the arithmetic average of the adequate values for blank. The standard deviation for each measurement was for the relative statement re-calculated as described above.

### Substrate specificity testing

Concentration of free inorganic phosphate released from different substrates was measured using the method described by Baginski, Epstein and Zak (1975) with minor alterations. An amount of 310 μl of 10 mM substrate in corresponding buffer (50 mM TRIS-HCl pH 8.3 for CaPho15p or 8.0 for CpPho15p, 10 mM MgCl_2_) was added to ∼100 μg of immobilised protein. The same amount of the chosen substrate was also added to 20 μl of protein-free matrix washed in Wash Buffer (10 mM imidazole, dH_2_O). These served as blanks. The mixture was incubated at 37°C on a rotator for 20 min. After the incubation, samples and blanks were centrifuged (45 s, 9960 g) and the supernatant was collected. The concentration of the free inorganic phosphate was estimated as described by Baginski, Epstein and Zak (1975) with minor alterations. Instead of arsenite citrate, sodium citrate was used. The working standards included values 0, 0.01, 0.05, 0.1 and 1 mmol of phosphate per litre. The blanks were prepared as described above, and the reagents were added to the blanks in the same order as for the samples.

### Mass spectrometry analysis of bacterially expressed phosphatases

The SDS-PAGE gels containing samples of CaPho15p and CpPho15p were prepared for mass spectrometry as described by Shevchenko *et al.* (2007) with slight modifications. Briefly, positive bands were excised and destained in 50% (v/v) acetonitrile (ACN) in 100 mM NH_4_HCO_3_ at room temperature for 30 min a dehydrated with pure ACN. Proteins were reduced in 10 mM dithiothreitol at 60°C for 30 min, dehydrated with pure ACN and then alkylated in 55 mM freshly prepared iodoacetamide at RT for 30 min in dark. After alkylation and dehydration, gel pieces were covered with trypsin solution (10 ng/μl in 10 mM NH_4_HCO_3_ containing 10% of ACN) for 30 min at 4°C and then incubated overnight at 37°C. Supernatant was collected, acidified with 5% trifluoroacetic acid and stored. Peptides from the gel were extracted with 60% ACN/2% formic acid at 37°C for 15 min. Extracted samples were dried and stored for further analysis.

The extracted samples from in-gel digestion were desalted using microcolumns prepared from GELoader tips with reverse-phase POROS® OLIGO™ R3 (Life Technologies) according to Gobom *et al.* (1999). Samples were directly eluted onto the MALDI plate using solution of α-cyano-4-hydroxycinnamic acid matrix (5 g/l in 60% ACN/0.1% TFA with 2 mM diammonium hydrogen citrate) which serves as a matrix. Mass spectrometric analysis of desalted samples was performed on a Thermo Scientific MALDI LTQ Orbitrap XL mass spectrometer (Thermo Fisher Scientific) and acquired in the positive mode with full MS scan (700–4000 m/z) at 60 000 FWHM at m/z 400.

### Mass spectrometry analysis of protein dephosphorylation

Phosphopeptides were dephosphorylated using 20 μl of settled Ni-NTA resin with Pho15p bound to via the His-tag. The resin was resuspended in 130 μl of a 50 mM Tris-HCl buffer (pH 8.3 or 8.0 for CaPho15p and CpPho15p, respectively) containing either 10 mM MgCl_2_, 1 mM CaCl_2_ or 1 mM MnCl_2_. The sample, 30 pmol of Phosphopeptide Standard Mixture (P33357, Thermo Fisher Scientific), containing peptides phosphorylated on serine, threonine and tyrosine. This mixture was incubated at 37°C under stirring. For analysis, 20 μl of supernatant was collected after 30, 120 and 300 min of incubation and the reaction was immediately terminated by addition of 3 μl of 5% trifluoroacetic acid. Samples were desalted and analysed using mass spectrometry as described in previous section, differing only in utilising solution of 2,5-dihydroxybenzoic acid (10 g/l in 50% ACN/0.1% TFA with 1% H_3_PO_4_) as MALDI matrix suitable for phosphopeptides (Larsen *et al.*^2005^).

### *K*_m_ determination

For the *K*_m_ determination, dilution series of the samples were prepared. The measurements were carried out as described above. The *K*_m_ values were calculated using SigmaPlot (Systat Software Inc.).

## RESULTS

### *In silico* analysis of PHO15 genes and construction of expression vectors

**Figure 1.**
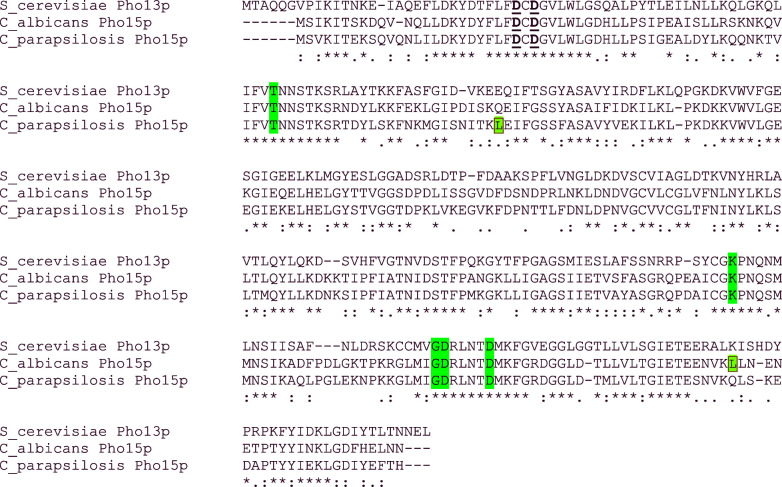
Sequence alignment of alkaline phosphatase Pho13p from *S. cerevisiae* and phosphatases Pho15p from *C. albicans* and *C. parapsilosis*. Aspartate residues of DxD signature are bold and underlined. Residues constituting motifs II, III and IV are highlighted in green. Leucines encoded by the ambiguous CTG codon are boxed and highlighted in blue.

The predicted alkaline phosphatases CaPho15p and CpPho15p share 75.4% identity and 86.4% similarity. Their identity to *S. cerevisiae* homologue ScPho13p is 51.2% for CaPho15p and 49.5% for CpPho15p (Fig. 1). The sequences of CaPho15p and CpPho15p differ mostly in the central part of the molecules and at the very C-termini. The regions in the proximity of the motifs I-IV are conserved.

*In silico* analysis using SignalP has not revealed signal sequences in these proteins (Petersen *et al.*^2011^). Analysis using WoLF PSORT indicates cytosolic localisation of CpPho15p but it also revealed a DNA-binding motif and a nuclear localisation signal. Nuclear localisation is even more likely in the case of CaPho15p (Horton *et al.*^2007^). The hallmark motifs of HAD superfamily are conserved among phosphatases from *Candida species* and from *S. cerevisiae*.

In order to express the *Candida PHO15* genes in *E. coli*, the whole coding sequences were amplified and inserted into pET28b, so that the resulting proteins have His-tags at the N-termini. Both *CaPHO15* and *CpPHO15* contain one CUG codon which encodes leucine in a standard genetic code. In *Candida* species belonging to the so-called CUG clade (or CTG clade), CUG is translated ambiguously, as serine or leucine. However, frequency of serine is ∼97% (Bezerra *et al.*^2013^). In pET28-albi-Pho15 and pET28-para-Pho15, the CTG codons were not replaced with standard codons for serine. Therefore, the phosphatases expressed in *E. coli* using these vectors contain Leu in corresponding positions (Fig. 1).

### Bacterial expression and purification of Pho15p

Both CaPho15p and CpPho15p were expressed in *E. coli* BL21(DE3), either using one expression plasmid or in two-plasmid co-expression system with five chaperones. While the use of the sole expression plasmids led to accumulation of phosphatases in inclusion bodies, during co-expression with chaperones the phosphatases remained soluble in cytosol. This, however, did not alleviate problems with solubility of both phosphatases. During the attempts to purify them from bacterial cytosol, both CaPho15p and CpPho15p precipitated and rapidly lost their activity.

**Figure 2.**
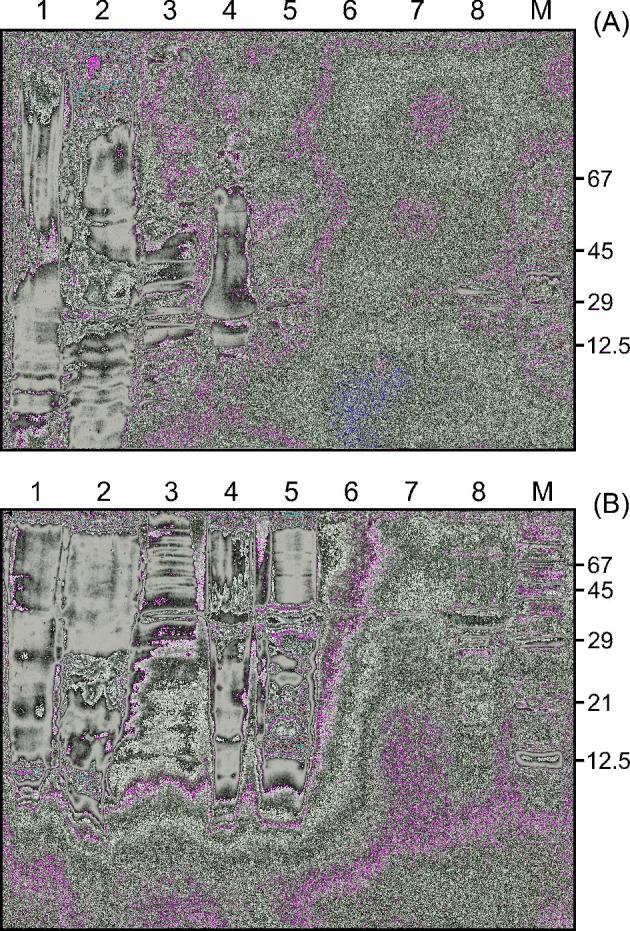
Bacterial expression and purification of CaPho15p (**A**) and CpPho15p (**B**). SDS-PAGE shows the protein composition of cell lysate (lane 1), soluble cytoplasmic fraction (lane 2), wash of the inclusion bodies (lane 3), inclusion bodies (lane 4), solubilised inclusion bodies after dialysis (lane 5), flow-through fraction after His-tagged protein binding to Ni-NTA Agarose (lane 6), wash of the column (lane 7), purified phosphatase (lane 8). M—Molecular weight marker.

Both enzymes were successfully purified from solubilised inclusion bodies. The highest yield of phosphatases was obtained when the cells were harvested 3 h post induction. Solubilisation of the inclusion bodies in 60% acetic acid and subsequent dialysis against water was the only way to obtain soluble protein samples suitable for further purification, which was performed using Ni-NTA Agarose (Fig. 2). Identity of both phosphatases was verified using mass spectrometry analysis (data not shown).

Several protocols were tested to release the phosphatases from the Ni-NTA Agarose, including elution with imidazole solution, with buffer of a high pH, or cleavage of the His-tag using thrombin. However, free phosphatases rapidly precipitated under all the conditions tested. Enzyme activity and substrate specificity testing was therefore performed using Pho15 enzymes attached to the Ni-NTA Agarose via His-tag. This setup enabled to preserve enzyme activity, which remained unchanged for several months.

### Dephosphorylation of para-nitrophenylphosphate by CaPho15p and CpPHO15p

**Figure 3.**
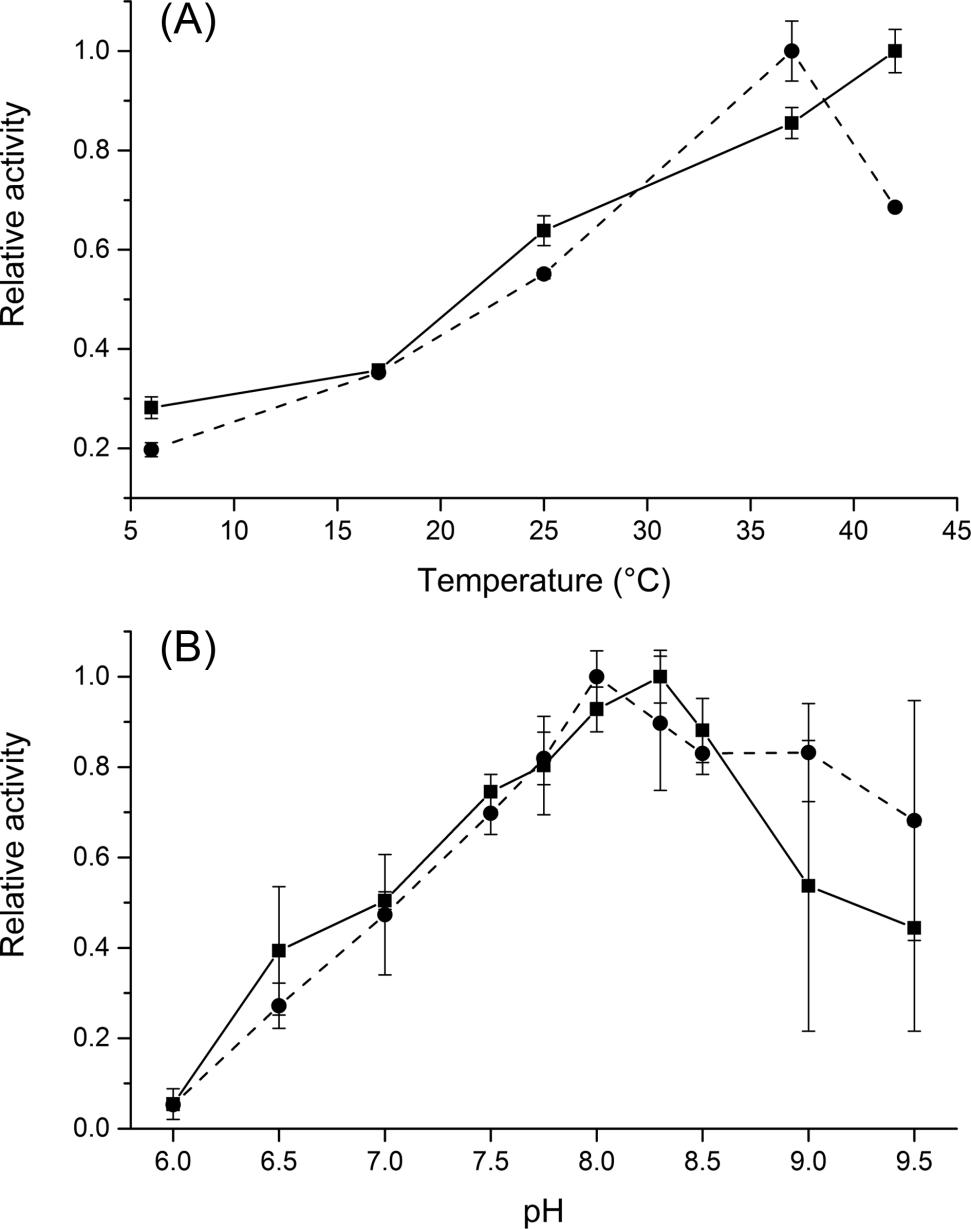
Effect of temperature (**A**) and pH (**B**) on the activity of CaPho15p (solid line) and CpPho15p (dashed line). The reactions were performed in triplicate, using immobilised phosphatases and pNPP as a substrate. The details are described in the Material and Methods section.

**Figure 4.**
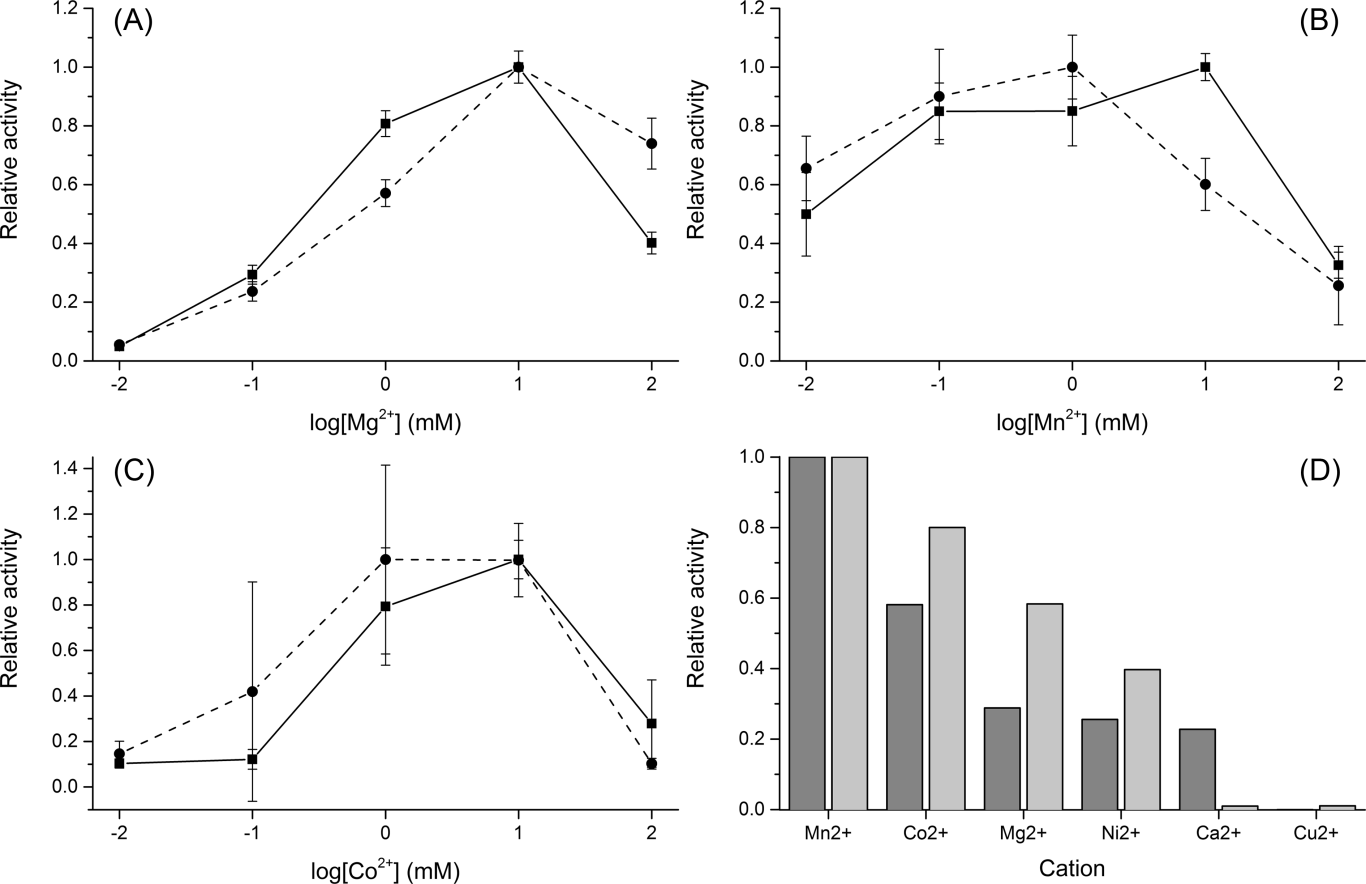
Effect of magnesium (**A**), manganese (**B**) and cobalt (**C**) on the activity of CaPho15p (solid line) and CpPho15p (dashed line). The measurements were performed in triplicate, using immobilised phosphatases and pNPP as a substrate. The procedure is described in the Materials and Methods section. Comparison of CaPho15p (dark bars) and CpPho15p (light bars) activities in the presence of different divalent cations (**D**). For this comparison, 10 mM concentration of each cation was used.

Both phosphatases readily hydrolysed pNPP. As illustrated by Fig. 3, pNPP was dephosphorylated most efficiently at pH 8–8.3 and at 42°C and 37°C by CaPho15p and CpPho15p, respectively. Both CaPho15p and CpPho15p required the presence of a divalent cation for their activity and were able to use different cations as cofactors, similarly as *S. cerevisiae* HAD-family phosphatases (Kuznetsova *et al.*^2015^). Although CaPho15p and CpPho15p showed slightly different profiles of optimal cofactor concentration (Fig. 4A–C), pNPP hydrolysis by both enzymes was supported by the cations in a similar order of efficiency: Mn^2+^ > Co^2+^ > Mg^2+^ > Ni^2+^ (Fig. 4D). CaPho15p was also active in the presence of Ca^2+^. Cu^2+^ was accepted as a cofactor by neither of the phosphatases.

### CaPho15p and CpPHO15p do not display desulphurylation activity

HAD family hydrolases are known to display desulphurylation activity. Therefore, hydrolysis of para-nitrophenylsulphate by CaPho15p and CpPho15p was tested. The experiments were performed under the optimum conditions required for pNPP dephosphorylation, either in the presence of Mg^2+^ or Mn^2+^, but no desulphurylation activity was detected.

### CaPho15p and CpPHO15p do not dephosphorylate phosphopeptides

As CaPho15p and CpPho15p were predicted to be alkaline phosphatases, phosphopeptides could be their natural substrates. For this purpose, Phosphopeptide Standard Mixture (P33357, Thermo Fisher Scientific), containing phosphorylated peptides at three different aminoacids (DHTGFLpTEpYVATR, TRDIpYETDYYRK, VPIPGRFDRRVpTVE and DLDVPIPGRFDRRVpSVAAE), was used as potential substrates. After incubation with CaPho15p and CpPho15p immobilised on Ni-NTA Agarose, no dephosphorylation was detected using mass spectrometry on either of phosphoserine, phosphothreonine or phosphotyrosine even after 300 min under optimised conditions.

### Substrate specificity of CaPho15p and CpPHO15p

**Figure 5.**
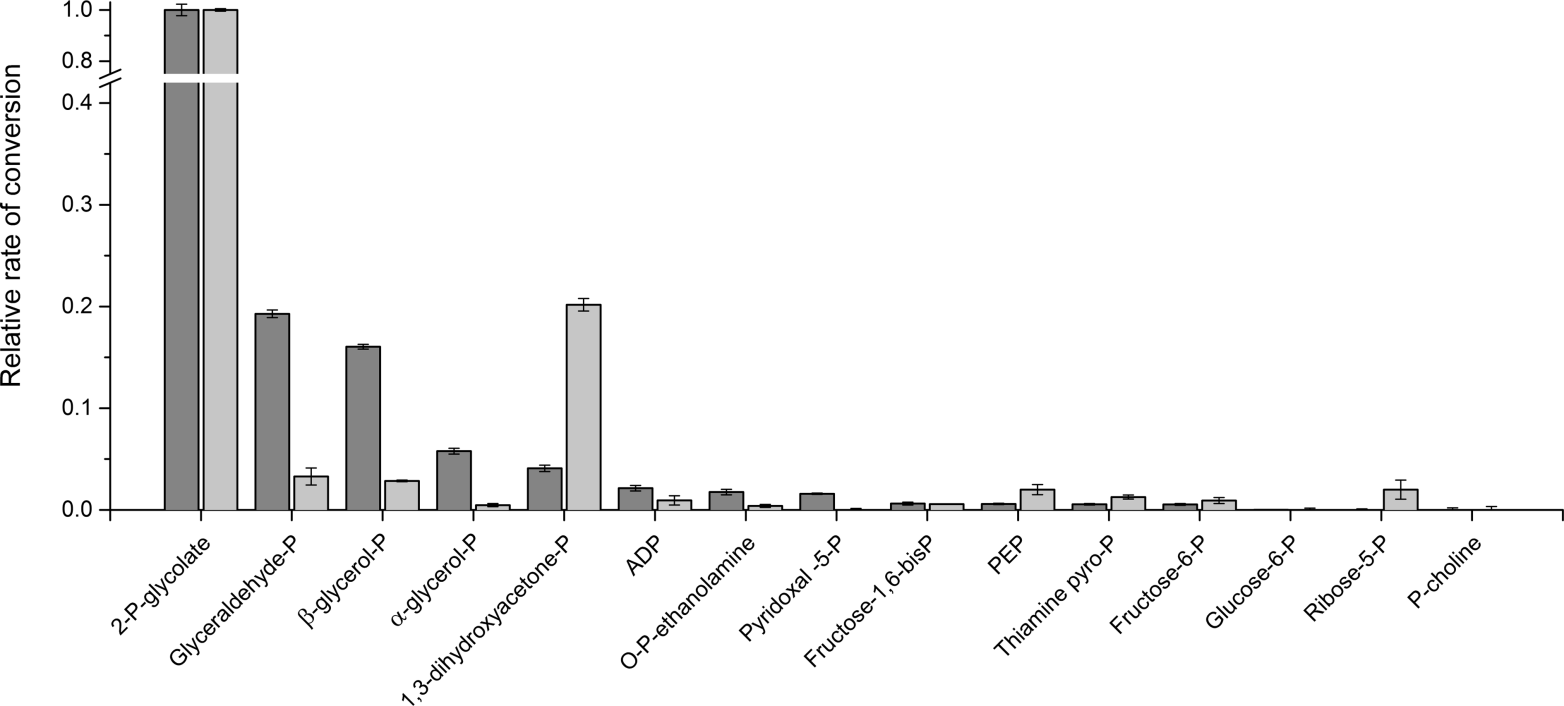
Comparison of substrate preferences of CaPho15p (dark bars) and CpPho15p (light bars). 2-P-glycolate, 2-phosphoglycolace; glyceraldehyde-P, glyceraldehyde-3-phosphate; β-glycerol-P, β-glycerophosphate; α-glycerol-P, α-glycerophosphate; 1,3-dihydroxyacetone-P, 1,3-dihydroxyacetone phosphate; ADP, adenosine diphosphate; O-P-ethanolamine, O-phosphorylethanolamine; pyridoxal-5-P, pyridoxal-5-phosphate; fructose-1,6-bisP, fructose-1,6-bisphosphate; PEP, phosphoenolpyruvate; thiamine pyro-P, thiamine pyrophosphate; fructose-6-P, fructose-6-phosphate; glucose-6-P, glucose-6-phosphate; ribose-5-P, ribose-5-phosphate; P-choline, phosphocholine.

**Figure 6.**
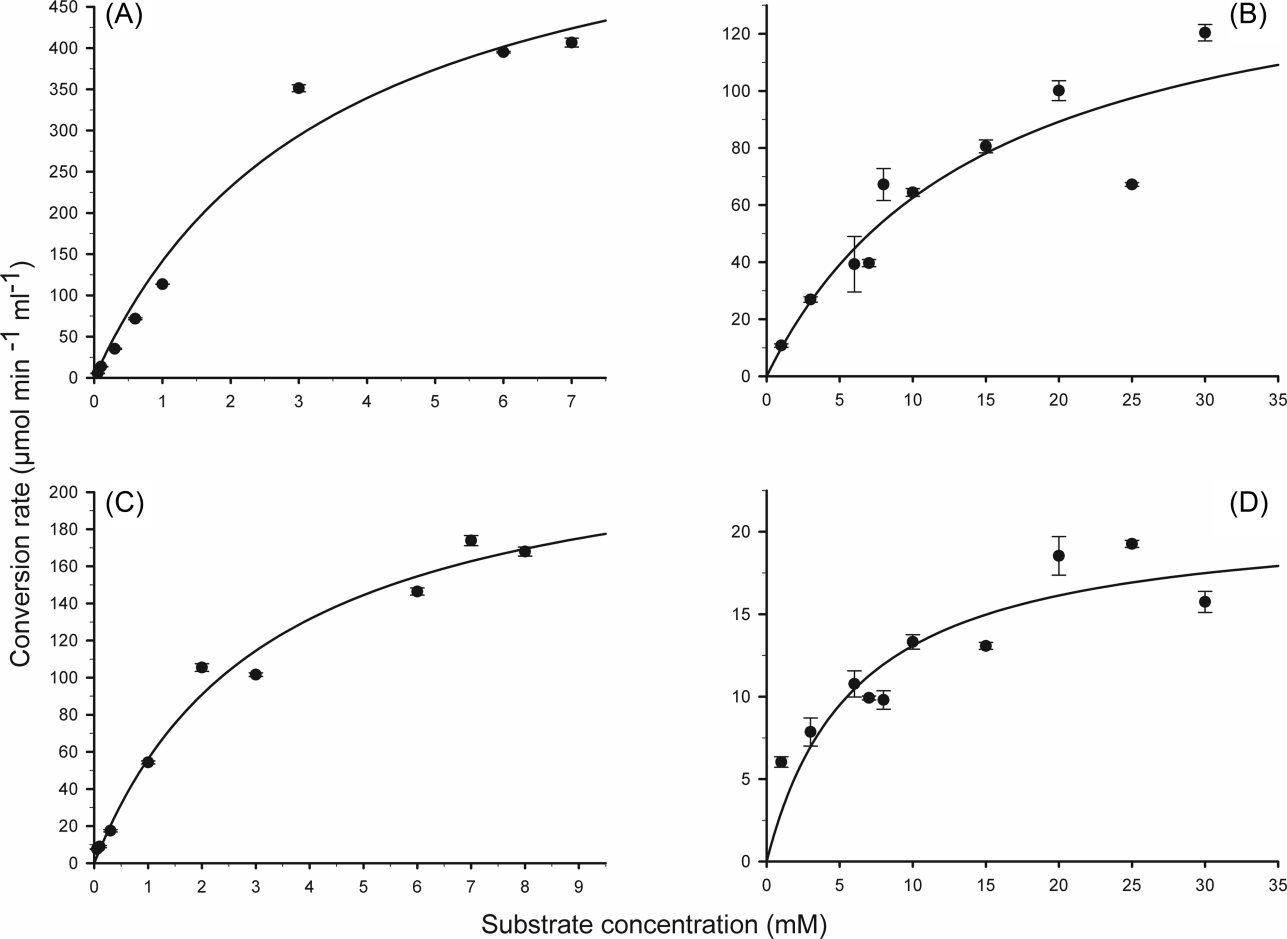
*K*_m_ graphs of CaPho15p (**A**, **B**) and CpPho15p (**C**, **D**) for 2-P-Glycolate (A, C) and 2-Glycerolphosphate (B, D). The *K*_m_ and *V*_max_ values are shown in Table 1. The measurements were performed in triplicate, using immobilised phosphatases and corresponding substrate. The procedure is described in the Materials and Methods section.

To identify potential natural substrates of CaPho15p and CpPho15p, a set of small phosphorylated molecules was selected for dephosphorylation testing. Both phosphatases hydrolysed most efficiently 2-phosphoglycolate, but differed in preferences for other substrates (Fig. 5). While CaPho15p dephosphorylated readily glyceraldehyde-3-phosphate and β-glycerol phosphate, CpPho15p preferred dihydroxyacetone phosphate. Phosphoenolpyruvate, phosphorylated sugars, ADP, pyridoxal phosphate, thiamine pyrophosphate and phosphoethanolamine were poor substrates or were not dephosphorylated at all. *K*_m_ values for 2-p-glycolate and 2-glycerolphosphate are in millimolar range for both phosphatases (Table 1, Fig. 6).

**Table 1.** *K*_m_ and *V*_max_ values for dephosphorylation of 2-phosphoglycolate and 2-glycerolphosphate by CaPho15p and CpPho15p.

	2-P-Glycolate		2-Glycerolphosphate	
	*K*_m_	*V*_max_	*K*_m_	*V*_max_
	mM	μmol min^−1^ ml^−1^	mM	μmol min^−1^ ml^−1^
CaPho15p	3.47 ± 0.6	634 ± 48	14.48 ± 4.3	155 ± 22
CpPho15p	3.26 ± 0.4	238 ± 12	6.07 ± 3.3	21 ± 18

Although hydrolysis of pNPP proceeded most efficiently in the presence of Mn^2+^ for both phosphatases, activity towards potentially natural substrates differed. Preferences for Mn^2+^ or Co^2+^ varied depending on a particular substrate–enzyme combination (data not shown). Mg^2+^ was therefore chosen because it is the biologically most relevant intracellular divalent cation and because it supported activity of both phosphatases similarly.

## DISCUSSION

Molecular function of the *PHO15* genes of *C. albicans* and *C. parapsilosis*, as listed in the *Candida* Genome Database, was inferred from electronic annotation, on the basis of homology to *S. cerevisiae PHO13* gene (www.candidagenome.org, 21 June 2018). However, the data on *PHO13* reported in literature are contradictory. Tuleva, Vasileva-Tonkova and Galabova (1998) published isolation and characterisation of authentic Pho13p phosphatase from *S. cerevisiae*, showing its histone dephosphorylation activity. Thus, when *C. albicans Δpho15* deletion strain displayed alterations in epigenetically regulated phenotypic feature known as white-opaque switching, it has been assumed to occur due to altered histone modification (Hnisz, Schwarzmüller and Kuchler 2009).

Nevertheless, *S. cerevisiae* Pho13p phosphatase has later been identified as a HAD-family protein; it was prepared by bacterial expression and characterised within a large set of the yeast HAD enzymes (Kuznetsova *et al.*^2015^). Recombinant Pho13p hydrolysed phosphorylated carbohydrates rather than phosphopeptides. This was in accordance with the results published by Collard *et al.* (2016), who described Pho13p as a homolog of mammalian phosphoglycolate phosphatase. These enzymes were found to be a part of the metabolic repair system thanks to their ability to hydrolyse phosphorylated side products of carbohydrate metabolism. The present work therefore mainly addressed the question, whether *C. albicans* and *C. parapsilosis* homologs of Pho13p dephosphorylate phosphopeptides or phosphorylated carbohydrates. The results clearly demonstrate that the latter is true. No dephosphorylation of phosphopeptides by CaPho15p and CpPho15p was detected, and the both phosphatases acted preferentially on 2-phosphoglycolate. Nonetheless, it should be noted that the experiments were performed with purified, immobilised enzymes and that the CUG codons have not been replaced with non-ambiguous serine codons. Each phosphatase thus contains one leucine residue at the position, which, under the natural conditions, is most probably occupied by serine. Although it is unlikely that these features profoundly change the substrate specificities of the Pho15p enzymes, minor alterations cannot be ruled out. Availability and usage of different cofactors may also affect the performance of the phosphatases studied.

The origin of 2-phosphoglycolate is well described in plants and cyanobacteria, where it is formed as a side product of ribulose-1,5-bisphosphate carboxylase/oxygenase and further metabolised by phosphoglycolate phosphatases producing glycolate and inorganic phosphate. 2-Phosphoglycolate is toxic, since it inhibits triosephosphate isomerase or phosphofructokinase (Kelly and Latzko 1976; Norman and Colman 1991). But it is also a signal molecule playing a role in acclimation to low CO_2_ concentrations and its intracellular level has to be controlled (Haimovich-Dayan *et al.*^2015^). The role of 2-phosphoglycolate in yeast and mammalian cells is not well defined. It is a side product of pyruvate kinase, which is formed from glycolate (Collard *et al.*^2016^). It is also generated as a result of DNA repair (Pellicer *et al.*^2003^). Nuclear localisation of Pho15p enzymes, which was suggested by the WoLF PSORT algorithm besides the presence in cytosol, would thus make sense.

While 2-phosphoglycolate was the best substrate for both phosphatases, CaPho15p and CpPho15p differed in preferences for other compounds tested. CpPho15p hydrolysed dihydroxyacetone phosphate, an intermediate of glycolysis pathway, which is formed from fructose-1,6-bisphosphate and converted to glyceraldehyde-3-phosphate. Triosephosphate isomerase catalysing reversible interconversion between dihydroxyacetone phosphate and glyceraldehyde-3-phosphate was found to be inhibited by 2-phosphoglycolate in plants (Norman and Colman 1991). Activity of CpPho15p towards 2-phosphoglycolate and dihydroxyacetone phosphate may thus indicate that this enzyme participates in regulation and scavenging of the glycolytic pathway reactions from fructose-1,6-bisphosphate to 1,3-bisphosphoglycerate in *C. parapsilosis*. Dephosphorylation of dihydroxyacetone phosphate yields dihydroxyacetone, which was found to be toxic for *S. cerevisiae*. Its toxicity, however, depends on a carbon source and can be suppressed by glucose (Molin, Norbeck and Blomberg 2003). This suggests potential role of CpPho15p in adaptation to changing carbon sources available in different host or environmental niches. The type of carbon source was found to influence cell wall properties and remodelling. Metabolic adaptation to available nutrients is often interrelated with expression of virulence factors (Brown *et al.*^2014^).

CaPho15 acts on ß-glycerophosphate, as the second best substrate of the compounds tested. Its dephosphorylation yields glycerol, which is important for osmoregulation and stress response. Contribution to increase of intracellular glycerol concentration may be one of the reasons why *CaPHO15* is upregulated under the stress conditions. Metabolic adaptation to stress conditions is intimately linked to virulence, as it increases the chance of *Candida* to survive within the host phagocytic cells. *CaPHO15* gene is one of ∼30 genes involved in the core stress response of *C. albicans*. Core stress response of *S. cerevisiae* is, in contrast, constituted by more than 200 genes, and *PHO13* in not among them (Brown *et al.*^2014^). This indicates that the orthologous phosphatases may play different physiological roles in these yeast species.

Pho13p from *S. cerevisiae* was found to hydrolyse 4-phospho-erythronate, a side product of glyceraldehyde-3-phosphate dehydrogenase and an inhibitor of 6-phospho-gluconate dehydrogenase. This important reaction enables coexistence of glycolysis and pentose phosphate pathway (Collard *et al.*^2016^). It has yet to be examined whether this function is conserved also for *Candida* Pho15p enzymes.

Recombinant phosphatases of two *Candida* species were rather difficult to work with due to their tendency to precipitate. Examination of the substrate specificities of CaPho15p and CpPho15p was made possible only thanks to the attachment of the enzymes to Ni-NTA Agarose via His-tag. *Candida albicans* and *C. parapsilosis* phosphatases hydrolyse 2-phosphoglycolate and 2-glycerolphosphate with a *K*_m_ one order of magnitude higher than Pho13p from *S. cerevisiae* (Collard *et al.*^2016^). These differences, which may, in part, be due to the different method of measurement, presence of the His-tag and enzyme immobilisation, are not likely to affect the overall picture of *Candida* Pho15p substrate specificity.

In conclusion, Pho15 phosphatases from *C. albicans* and *C. parapsilosis* are HAD-family 2-phosphoglycolate phosphatases. They may be a part of metabolic repair system, but their precise role in *Candida* spp. physiology has yet to be investigated.

## Notes

**Present address:** Department of Epidemiology, Faculty of Military Health Sciences, University of Defence, Hradec Králové.

## References

[bib1] Baginski ES, Epstein E, Zak B Review of phosphate methodologies. Ann Clin Lab Sci 1975;5:399–416. 1180482

[bib2] Berman J. *Candida albicans*. Curr Biol 2012;22:R620–2. 22917504 10.1016/j.cub.2012.05.043

[bib3] Bezerra AR, Simões J, Lee W Reversion of a fungal genetic code alteration links proteome instability with genomic and phenotypic diversification. Proc Natl Acad Sci USA 2013;110:11079–84. 23776239 10.1073/pnas.1302094110 PMC3704024

[bib4] Bilir SP, Ferrufino CP, Pfaller MA The economic impact of rapid *Candida* species identification by T2 *Candida* among high-risk patients. Future Microbiol 2015;10:1133–44. 25848692 10.2217/fmb.15.29

[bib5] Bondaryk M, Kurzątkowski W, Staniszewska M Antifungal agents commonly used in the superficial and mucosal candidiasis treatment: mode of action and resistance development. Adv Dermatol Allergol Dermatol Alergol 2013;30:293–301. 10.5114/pdia.2013.38358 PMC3858657 24353489

[bib6] Brown AJP, Budge S, Kaloriti D Stress adaptation in a pathogenic fungus. J Exp Biol 2014;217:144–55. 24353214 10.1242/jeb.088930 PMC3867497

[bib7] Burroughs AM, Allen KN, Dunaway-Mariano D Evolutionary genomics of the HAD superfamily: understanding the structural adaptations and catalytic diversity in a superfamily of phosphoesterases and allied enzymes. J Mol Biol 2006;361:1003–34. 16889794 10.1016/j.jmb.2006.06.049

[bib8] Butler G, Rasmussen MD, Lin MF Evolution of pathogenicity and sexual reproduction in eight *Candida* genomes. Nature 2009;459:657–62. 19465905 10.1038/nature08064 PMC2834264

[bib9] Casadevall A, Pirofski L What is a host? Incorporating the microbiota into the damage-response framework. Infect Immun 2015;83:2–7. 25385796 10.1128/IAI.02627-14 PMC4288903

[bib10] Collard F, Baldin F, Gerin I A conserved phosphatase destroys toxic glycolytic side products in mammals and yeast. Nat Chem Biol 2016;12:601–7. 27294321 10.1038/nchembio.2104

[bib11] Ding C, Vidanes GM, Maguire SL Conserved and divergent roles of Bcr1 and CFEM proteins in *Candida parapsilosis* and *Candida albicans*. PLoS One 2011;6:e28151. 22145027 10.1371/journal.pone.0028151 PMC3228736

[bib12] Enjalbert B, Smith DA, Cornell MJ Role of the Hog1 stress-activated protein kinase in the global transcriptional response to stress in the fungal pathogen *Candida albicans*. Mol Biol Cell 2006;17:1018–32. 16339080 10.1091/mbc.E05-06-0501 PMC1356608

[bib13] Gobom J, Nordhoff E, Mirgorodskaya E Sample purification and preparation technique based on nano-scale reversed-phase columns for the sensitive analysis of complex peptide mixtures by matrix-assisted laser desorption/ionization mass spectrometry. J Mass Spectrom 1999;34:105–16. 10093212 10.1002/(SICI)1096-9888(199902)34:2<105::AID-JMS768>3.0.CO;2-4

[bib14] Guinea J Global trends in the distribution of *Candida* species causing candidemia. Clin Microbiol Infect 2014;20:5–10. 10.1111/1469-0691.12539 24506442

[bib15] Haimovich-Dayan M, Lieman-Hurwitz J, Orf I Does 2-phosphoglycolate serve as an internal signal molecule of inorganic carbon deprivation in the cyanobacterium *Synechocystis* sp. PCC 6803? Environ Microbiol 2015;17:1794–804. 25297829 10.1111/1462-2920.12638

[bib16] Hnisz D, Schwarzmüller T, Kuchler K Transcriptional loops meet chromatin: a dual-layer network controls white-opaque switching in *Candida albicans*. Mol Microbiol 2009;74:1–15. 19555456 10.1111/j.1365-2958.2009.06772.x PMC2764112

[bib17] Hoffman CS, Winston F A ten-minute DNA preparation from yeast efficiently releases autonomous plasmids for transformation of *Escherichia coli*. Gene 1987;57:267–72. 3319781 10.1016/0378-1119(87)90131-4

[bib18] Horton P, Park K-J, Obayashi T WoLF PSORT: protein localization predictor. Nucleic Acids Res 2007;35:W585–7. 17517783 10.1093/nar/gkm259 PMC1933216

[bib19] Jones T, Federspiel NA, Chibana H The diploid genome sequence of *Candida albicans*. Proc Natl Acad Sci USA 2004;101:7329–34. 15123810 10.1073/pnas.0401648101 PMC409918

[bib20] Kelly GJ, Latzko E Inhibition of spinach-leaf phosphofructokinase by 2-phosphoglycollate. FEBS Lett 1976;68:55–58. 134908 10.1016/0014-5793(76)80403-6

[bib21] Kuznetsova E, Nocek B, Brown G Functional diversity of haloacid dehalogenase superfamily phosphatases from *Saccharomyces cerevisiae*: biochemical, structural, and evolutionary insights. J Biol Chem 2015;290:18678–98. 26071590 10.1074/jbc.M115.657916 PMC4513125

[bib22] Larsen MR, Thingholm TE, Jensen ON Highly selective enrichment of phosphorylated peptides from peptide mixtures using titanium dioxide microcolumns. Mol Cell Proteomics 2005;4:873–86. 15858219 10.1074/mcp.T500007-MCP200

[bib23] Molin M, Norbeck J, Blomberg A Dihydroxyacetone kinases in *Saccharomyces cerevisiae* are involved in detoxification of dihydroxyacetone. J Biol Chem 2003;278:1415–23. 12401799 10.1074/jbc.M203030200

[bib24] Motosugi K, Esaki N, Soda K Purification and properties of a new enzyme, DL-2-haloacid dehalogenase, from *Pseudomonas* sp. J Bacteriol 1982;150:522–7. 7068529 10.1128/jb.150.2.522-527.1982 PMC216397

[bib25] Norman EG, Colman B Purification and characterization of phosphoglycolate phosphatase from the Cyanobacterium *Coccochloris peniocystis*. Plant Physiol 1991;95:693–8. 16668041 10.1104/pp.95.3.693 PMC1077593

[bib26] O’Brien PJ, Herschlag D Alkaline phosphatase revisited: hydrolysis of alkyl phosphates. Biochemistry 2002;41:3207–25. 11863460 10.1021/bi012166y

[bib27] Pabis A, Kamerlin SCL Promiscuity and electrostatic flexibility in the alkaline phosphatase superfamily. Curr Opin Struct Biol 2016;37:14–21. 26716576 10.1016/j.sbi.2015.11.008

[bib28] Pellicer MT, Nuñez MF, Aguilar J Role of 2-phosphoglycolate phosphatase of *Escherichia coli* in metabolism of the 2-phosphoglycolate formed in DNA repair. J Bacteriol 2003;185:5815–21. 13129953 10.1128/JB.185.19.5815-5821.2003 PMC193966

[bib29] Petersen TN, Brunak S, Heijne G von SignalP 4.0: discriminating signal peptides from transmembrane regions. Nat Methods 2011;8:785–6. 21959131 10.1038/nmeth.1701

[bib30] Pfaller MA, Castanheira M Nosocomial candidiasis: antifungal stewardship and the importance of rapid diagnosis. Med Mycol 2016;54:1–22. 26385381 10.1093/mmy/myv076

[bib31] Polke M, Hube B, Jacobsen ID *Candida* survival strategies. Advances in Applied Microbiology 2015;91:139–235. 10.1016/bs.aambs.2014.12.002 25911234

[bib32] Sambrook J, Rusell DW Molecular Cloning: a Laboratory Manual. Cold Spring Harbor, NY, USA: Cold Spring Harbor laboratory Press, 2001.

[bib33] Seifried A, Schultz J, Gohla A Human HAD phosphatases: structure, mechanism, and roles in health and disease. FEBS J 2013;280:549–71. 22607316 10.1111/j.1742-4658.2012.08633.x

[bib34] Shevchenko A, Tomas H, Havli J In-gel digestion for mass spectrometric characterization of proteins and proteomes. Nat Protoc 2007;1:2856–60. 10.1038/nprot.2006.468 17406544

[bib35] Tuleva B, Vasileva-Tonkova E, Galabova D A specific alkaline phosphatase from *Saccharomyces cerevisiae* with protein phosphatase activity. FEMS Microbiol Lett 1998;161:139–44. 9561742 10.1111/j.1574-6968.1998.tb12940.x

